# Composition of Ileal Bacterial Community in Grazing Goats Varies across Non-rumination, Transition and Rumination Stages of Life

**DOI:** 10.3389/fmicb.2016.01364

**Published:** 2016-09-05

**Authors:** Jinzhen Jiao, Jian Wu, Chuanshe Zhou, Shaoxun Tang, Min Wang, Zhiliang Tan

**Affiliations:** ^1^Key Laboratory for Agro-Ecological Processes in Subtropical Region, Hunan Research Center of Livestock and Poultry Sciences, South Central Experimental Station of Animal Nutrition and Feed Science in the Ministry of Agriculture, Institute of Subtropical Agriculture, The Chinese Academy of SciencesChangsha, China; ^2^Hunan Co-Innovation Center of Animal Production SafetyCICAPS, Changsha, China; ^3^Graduate University of Chinese Academy of SciencesBeijing, China

**Keywords:** bacterial colonization, fermentation capacity, ileum, age, goats

## Abstract

The establishment of gut microbiota is increasingly recognized as a crucial action in neonatal development, host health and productivity. We hypothesized that the ileal microbiome shifted as goats matured, and this colonization process would be associated with host fermentation capacity. To this end, 18 Liuyang black grazing goats were randomly slaughtered at d 0, 7, 28, 42, and 70. Ileal microbiota was profiled by Miseq sequencing of 16S rRNA gene of bacteria, and fermentation capacity [volatile fatty acid, activities of amylase, carboxymethylcellulase (CMCase) and xylanase] was determined using digesta sample. Principal coordinate analysis revealed that each age group harbored its distinct bacteria. Total bacteria copy number and most alpha diversity indexes increased (*P* < 0.01) from d 0 to 70. At the phylum level, abundances of *Cyanobacteria* (*P* = 0.018) and *TM7* (*P* = 0.010) increased linearly, abundances of *Bacteroidetes* (*P* = 0.075) and *Fibrobacteres* (*P* = 0.076) tended to increase linearly, whist *Proteobacteria* abundance tended to decline quadratically (*P* = 0.052) with age. At the genus level, *Enterococcus* (30.9%), *Lactobacillus* (32.8%), and *Escherichia* (2.0%) dominated at d 0, while *Prevotella*, *Butyrivibrio*, *Ruminococcus*, *SMB53*, and *Fibrobacter* surged in abundance after day 20. The highest amylase activity was observed at day 42, while xylanase activity increased quadratically (*P* = 0.002) from days 28 to 70. Correlation analysis indicated that abundances of *Bacteroides*, *Clostridium*, *Lactobacillus*, *Propionibacterium, Enterococcus*, and *p-75-a5* positively correlated with enzyme activity. Collectively, ileal bacteria in grazing goats assemble into distinct communities throughout development, and might participate in the improvement of host fermentation capacity.

## Introduction

The mammal intestine resides 100 trillion microorganisms, with major bacterial phyla comprising of *Bacteroidetes*, *Firmicutes*, and *Proteobacteria* (Donaldson et al., 2016). Many intestinal commensals can promote various physiological functions in terms of nutrition, immunity and defense in normal mammals (Round and Mazmanian, 2009; Bernalier-Donadille, 2010). Using *in vitro* cultivation technique, our previous studies have suggested that except for the rumen and large intestine, the ileum also serves as an indispensable fermentation site in goats (Jiao et al., 2014a,b). Furthermore, Zeng et al. (2015) proposed that sheep ileum harbored a larger number of cellulolytic bacteria, particularly *Clostridium cluster IV* (10^8^ copies per gram digesta). Generally, anaerobic digestion of carbohydrate depends on a wide range of microbial groups, especially fibrolytic bacteria, and leads to the formation of volatile fatty acids (VFA), carbon dioxide (CO_2_), hydrogen (H_2_), and microbial biomass (Bernalier-Donadille, 2010).

Next generation sequencing technology has advanced our knowledge about intestinal microbial diversity and function. Using pyrosequencing, Malmuthuge et al. (2014) pointed out that in pre-weaned calves, the ileal digesta bacterial community contained primarily *Firmicutes* (97.7%), consisting of *Lactobacillus* (44.5%), *Clostridium* (16.7%), and *Sharpea* (8.9%). In goat ileum, barcoded DNA pyrosequencing revealed that the proportions of genera *Acetitomaculum*, *Enterococcus*, *Atopobium*, unclassified *Coriobacteriaceae*, and unclassified *Planctomycetaceae* were decreased in dietary treatment containing greater proportion of corn grain (50% vs. 25%, 0%) (Mao et al., 2013). This implies that the ileal bacterial community is diet-dependant.

During animal development process, ruminants undergo a drastic change in nutrient supply from high-fat milk diets during non-rumination phase to forage based diets during rumination phase. Since ruminants depend on rumen microbes to degrade plant lignocellulosic material, investigating colonization process of the rumen microbiome has become the research interest of several scientists. Rey et al. (2014) reported that rumen digesta bacteria community undergoes a three-stage implantation process with a progressive but important shift of composition from day 1 to weaning at day 83 of age. For goat rumen epithelial bacteria, *Escherichia* genus (80.79%) dominated at day 0, while *Prevotella*, *Butyrivibrio*, and *Campylobacter* genera surged in abundance at days 42 and 70 (Jiao et al., 2015b). Compared to considerable studies focusing on rumen microbial succession, few studies have attempted to investigate bacteria colonization process in ruminant ileum, especially in grazing animals. Therefore, the present study aimed to explore age-related changes in ileal bacterial community for grazing goats and their potential roles in host fermentation capacity.

## Materials and Methods

### Animals

Eighteen newly born Liuyang black goat kids (average weight of 1.35 ± 0.12 kg) were separated from the nanny, placed in individual pens, and trained to suckle milk from nipple pails. From days 0 to 20, kids were provided with goat milk. From days 20 to 40, the kids were provided with goat milk and grazed on pasture. After day 40, kids received no milk and just grazed on pasture. Three kids were slaughtered at days 0 and 7, and four kids were slaughtered at days 28, 42, and 70, respectively. Detailed feeding management, ingredients of forage (mainly twitch grass, *Miscanthus*) have been described in our previous parallel study (Jiao et al., 2015b). All experiments were approved and performed following the guidelines of the Animal Care Committee, Institute of Subtropical Agriculture, Chinese Academy of Sciences, Changsha, China.

### Sampling Procedures

The ileal digesta were collected immediately after slaughter. One gram digesta was snap-frozen in liquid nitrogen and kept frozen at -80°C until processing for DNA extraction. Three grams digesta were homogenized with 1 mL of 25% (wt/vol) metaphosphoric acid and 6 mL distilled water and then centrifuged (17,000 × *g* at 4°C for 10 min), and the supernatant was stored at -20°C for VFA analysis. Three grams digesta were diluted 1:3 (wt:vol) with pre-warmed (39°C), anaerobic, sterile buffer (0.1 M citrate-phosphate buffer, pH 6.6) and stored at -20°C for assay of enzyme activity.

### Chemical Analysis

Volatile fatty acids were assayed from chromatograph peak areas using calibration with external standards using a gas chromatograph (7890A, Agilent, Wilmington, DE, USA). The VFA were separated with a nitroterephthalic acid modified polyethylene glycol column (30 m × 250 um × 0.25 um) with 0.8 mL/min nitrogen gas flow rate, and detected with a flame ionization detector. Amylase, carboxymethylcellulase (CMCase), and xylanase activities were assayed according to the methods detailed by Jiao et al. (2014b), using starch, carboxymethylcellulose (CMC), and xylan (all purchased from Sigma Chemicals, St. Louis, MO, USA) as substrates, respectively. Briefly, rumen digesta samples were thawed at 4°C, and submitted to ultrasonic disintegration for three cycles for 15 s with 10-s intervals at 4°C on a Vibra CellTM sonicator (Bertin technologies, Montigny le Bretonneux, France). The samples were centrifuged (20,000 × *g* for 15 min at 4°C) to separate cellular debris and enzymes, and only the supernatants were used to assay enzyme activities. The enzyme reactions were initiated by addition of 0.5 mL enzyme solution into a tube containing a mixture of 1 mL substrate and 0.5 mL citrate phosphate buffer, which were prior incubated at 39°C for 10 min. The reaction times for amylase, CMCase, and xylanase were 15, 30, and 15 min, respectively. One enzyme activity unit (U) was defined as the amount of enzyme required to release 1 mmol of xylose or glucose equivalents per min per g of wet ileal content.

### DNA Extraction and Quantification of Total Bacteria

The genomic DNA was extracted from the ileal digesta samples using the QIAamp DNA Stool Mini Kits (Qiagen GmbH, Hilden, Germany). Absolute quantitative real-time PCR was performed to quantify copy numbers of 16S rRNA genes of total bacteria according to the methods detailed in our previous study (Jiao et al., 2015b). The copy number of total bacteria 16SrRNA gene per gram of fresh digesta was calculated as according to the formula suggested by Zhou et al. (2009). The copy numbers were converted to log10 for further statistical analysis.

### PCR and High-Throughput Sequencing

The extracted DNA was subjected to PCR amplification of the V2 and V3 region of 16S rRNA gene using universal primer 104F (5′-*NNNNNN* GGCGVACGGGTGAGTAA-3′) and 530R (5′-CCGCNGCNGCTGGCAC-3′) using procedures as described by Jiao et al. (2015a). The forward primer, 104F, contained 6-base barcodes represented by the italicized poly (N) section of the primer. The PCR products were purified using Wizard^®^ SV Gel and PCR Clean-Up System (Promega, Madison, USA). The amplicons generated for each sample was quantified using QuantiFluor^TM^ -ST system (Promega, Madison, WI, USA), and pooled in equimolar concentrations. The 16S rRNA library was prepared according to the instruction of Nextera XT Index kits (Illumina, San Diego, CA, USA). Sequencing was conducted on an Illumina MiSeq PE300 instrument, according to the procedures of Miseq reagent kits v3 (Illumina, San Diego, CA, USA).

### Data Analysis

The bacterial 16S rRNA reads were analyzed using QIIME (Caporaso et al., 2010) and mothur (Schloss et al., 2009) pipeline. Pair-end reads were deconvoluted into individual samples based on their barcodes and assembled base on their overlap sequence using Connecting Overlapped Pair-End (COPE; Liu et al., 2012). After primer trimming, the assembled sequences were then assigned to operational taxonomic units (OTUs) at 97% similarity using UPASE (Edgar, 2013). Sequence alignment was performed by PyNAST and phylogenetic tree was constructed using FastTree (Price et al., 2009). Taxonomic assignments within the latest Greengenes database (Greengenes May, 2013 release) were generated using the mothur-based implementation of the RDP Bayesian classifier with a 0.80 confidence threshold (DeSantis et al., 2006).

Alpha diversity of ileal bacterial communities of grazing kids at different ages was obtained using the alpha rarefaction pipeline. Principal coordinate analysis (PCoA) was performed using unweighted Unifrac distance. Transformation-based principal components analysis (tb-PCA) was applied to ordinate the 18 samples according to OTU abundance data (Legendre and Gallagher, 2001). Only the top 10 OTUs for which variation of abundance contributed most to the clustering obtained from the tb-PCA were selected and represented.

### Statistical Analysis

A power test using GPower software was performed on the major bacteria genera (*Lactobacillus*, *Ruminococcus* and *SMB53*), as well as on enzyme activity. The results indicated that the within-group variation is low, with four replicates reaching a power > 0.80 when comparing two ages.

The effect of age on ileal bacterial community and fermentation parameters was investigated using the MIXED procedures of SAS (SAS Inst. Inc., Cary, NC, USA). Tukey’s test was used to compare least squares means. The model included the fixed effect of age and the random effect of animal within age. Linear and quadratic effects of age were analyzed using orthogonal contrasts. Statistical significance was accepted at *P* < 0.05 and a trend was considered at *P* < 0.10. All presented data are expressed as least-squares means.

The PROC CORR procedure of SAS was used to determine the Spearman’s rank correlations between ileal bacterial community and fermentation parameters. Only bacterial genus that represented >1% of the total community in one sample at least and that were detected in >50% of the samples were included in the analysis.

### Nucleotide Sequence Accession Numbers

Sequencing data in this study were deposited in the NCBI Sequence Read Archive (SRA) under accession numbers SRR3524811 to SRR3524822, SRR3524899, SRR3524924, SRR3524946, SRR3524987, SRR3524998, and SRR3525010.

## Results

### Fermentation Parameters

The values for VFA and enzyme activities were not presented for the reason that they were not detected due to insufficient sample amount. Meanwhile, except for acetate other VFA (i.e., propionate and butyrate etc.) concentrations were not detectable at experimental period, which were thereby not listed in **Table 1**. Age exerted no effects on acetate concentration (*P* = 0.317, linearly) and CMCase activity (*P* = 1.000, linearly) (**Table 1**). Age exhibited quadratic effect on amylase activity (*P* < 0.001), while exerted quadratical increasing effect (*P* = 0.002) on xylanase activity from days 28 to 70.

**Table 1:** Volatile fatty acid (VFA) and enzyme activities of ileal contents at different ages in grazing kids.

Item	Age (d)					SEM	*P*-value	
	0^1^	7^1^	28	42	70		*L*	*Q*
Acetate, mmol	-	-	3.39	4.05	3.52	0.54	1.000	0.384
Amylase, *U*^2^	-	-	1.13	2.03	0.59	0.03	<0.001	<0.001
CMCase, *U*	-	-	0.08	0.10	0.10	0.02	0.317	0.429
Xylanase, *U*	-	-	0.18	0.29	0.27	0.02	0.008	0.002

*^1^The values for acetate and enzyme activities at days 0 and 7 were not presented for the reason that they were not detected due to insufficient sample amount.*

*^2^For enzyme activities, amylase, CMCase and xylanase activities were expressed as the amount of enzyme required to release 1 μmol of reducing sugars (xylose or glucose equivalent) per min per g of intestinal wet content*.

### Total Bacteria Copy Number

Age exerted a quadratic effect (*P* < 0.001) on total bacteria copy number, with the greatest value observed at day 28 (**Table 2**). Specifically, total bacteria copy number increased sharply (almost 500 times) during the 1st week, and its value also experienced another drastic increase at day 28 (almost 100 times), followed by a decline afterward.

**Table 2:** Total bacteria copy number and alpha diversity measures of ileal bacterial community at different ages in grazing kids.

Index	Age (d)					SEM	*P*-value	
	0	7	28	42	70		*L*	*Q*
Total bacteria^1^	5.63	8.32	10.40	9.19	9.58	0.209	<0.001	<0.001
OTU number	73.3	293.0	729.5	588.3	653.3	43.80	<0.001	<0.001
Chao	135.6	525.6	978.2	881.6	933.6	55.63	<0.001	<0.001
Ace	212.3	733.6	964.1	978.1	1021.4	80.81	<0.001	0.002
Shannon	1.13	2.13	4.43	4.12	3.94	0.327	<0.001	<0.001
Simpson	0.59	0.26	0.06	0.05	0.10	0.060	<0.001	0.001
Coverage	0.998	0.991	0.986	0.987	0.986	0.001	<0.001	<0.001

*^1^Total bacteria was expressed as log _10_ copy numbers per gram ileal wet digesta*.

### Sampling Depth and Alpha Diversity

Nearly 878,590 sequences were generated for ileal bacteria, with an average of 48,811 sequences for each sample (the minimum value was 16,694 and the maximum was 105,903). This yielded 3,114 representative OTUs based on 97% similarity. With subsample of 16,000 reads every sample, both the rarefaction curves (Supplementary Figure S1) and the high coverage value (from 0.986 to 0.998, **Table 2**) showed the sampling depth is enough. Age exhibited quadratic effect (*P* < 0.001) on OTU number, with the highest value detected at day 28. Chao, Ace and Shannon index increased quadratically (*P* < 0.001), while Simpson index and coverage declined quadratically (*P* < 0.001) from days 0 to 70 (**Table 2**).

### Taxonomic Composition of Ileal Bacterial Community

Twenty-two phyla were identified, with 12 phyla abundances were <0.5%, and only a small proportion of sequences (<1%) remained unclassified at the phylum level (**Table 3**). *Cyanobacteria* (*P* = 0.018) and *TM7* (*P* = 0.010) abundances increased linearly, *Bacteroidetes* (*P* = 0.075), *Fibrobacteres* (*P* = 0.076), and *Tenericutes* (*P* = 0.069) abundances tended to increase, whist *Proteobacteria* abundance tended to decline quadratically (*P* = 0.052) from days 0 to 70. *Actinobacteria* abundance in one sample at day 0 peaked at 9.96%, and *Verrucomicrobia* abundance in one sample at day 7 peaked at 11.51%, respectively. *Fusobacteria* was only detected at day 7 (5.47%). *Firmicutes* abundance fluctuated with age, whereas age had a quadratic effect on *Tenericutes* abundance (*P* = 0.007).

**Table 3:** Phylum level composition (% of sequences) of ileal bacterial community at different ages in grazing kids.

Phylum	Age (d)					SEM	*P*-value	
	0	7	28	42	70		*L*	*Q*


*Actinobacteria*	3.07	0.30	0.17	0.30	0.19	1.041	0.194	0.267


*Bacteroidetes*	0.39	6.29	11.63	5.70	11.83	3.289	0.075	0.580


*Cyanobacteria*	0.00	0.01	12.67	14.22	17.34	5.327	0.018	0.372


*Fibrobacteres*	0.00	0.01	0.63	0.71	0.83	0.357	0.076	0.478


*Firmicutes*	69.06	77.50	65.66	51.41	44.46	13.673	0.112	0.943


*Fusobacteria*	0.00	5.47	0.00	0.00	0.00	1.857	0.293	0.823


*Proteobacteria*	23.89	5.43	1.38	4.23	3.43	4.505	0.036	0.052


*TM7*	0.01	0.20	3.47	16.71	19.18	5.359	0.010	0.916


*Tenericutes*	0.00	0.03	2.50	5.63	1.41	1.036	0.069	0.007


*Verrucomicrobia*	0.00	3.86	0.01	0.03	0.01	1.300	0.295	0.829


Unclassified	0.57	0.53	0.18	0.04	0.14	0.257	0.172	0.355


Others (<0.5%)	0.01	0.37	1.70	1.02	1.18	0.424	0.074	0.100

A great proportion of sequences (9.86 to 66.90%) remained unclassified at the genus level. Within the *Bacteroidetes* phylum, *Bacteroides* abundance declined linearly (*P* = 0.049), whist *Prevotella* abundance increased linearly (*P* = 0.022) from days 0 to 70 (**Table 4**). Within the *Firmicutes* phylum, abundances of *Butyrivibrio*, *Coprococcus*, *Pseudobutyrivibrio*, and *Ruminococcus* increased linearly (*P* < 0.05), abundances of *Clostridium* (*P* = 0.091) and *Moryella* (*P* = 0.082) tended to increase linearly, whereas *Lactobacillus* abundance decreased linearly (*P* = 0.004) from days 0 to 70. Age exerted quadratic effects on abundances of *Mogibacterium* (*P* = 0.005) and *p-75-a5* (*P* = 0.002), and tended to have quadratic effects on abundances of *Bulleidia* (*P* = 0.095) and *SMB53* (*P* = 0.074). *Enterococcus* abundance peaked at 92.09% in one sample at day 0, and the highest *Oscillospira* abundance were 0.83% (day 70). Within the *Proteobacteria* phylum, *Escherichia* abundance fluctuated with age. Within the *Actinobacteria* phylum, abundances of *Bifidobacterium* (0.58%) and *Propionibacterium* (3.11%) peaked at one sample from day 0. Moreover, age exerted quadratic effect (*P* = 0.020) on *SHD-231* abundance, while *Fibrobacter* abundance tended to increase linearly (*P* = 0.076) from d 0 to 70. *Treponema* and *Akkermansia* abundances peaked at 0.61% (day 28) and 3.86% (day 7), respectively.

**Table 4:** Genus level composition (% of sequences) of ileal bacterial community at different ages in grazing kids.

Phylum	Genus	Age (d)					SEM	*P*-value	
		0	7	28	42	70		*L*	*Q*
*Bacteroidetes*	*Bacteroides*	0.04	1.61	0.01	0.14	0.15	0.223	0.049	0.536


	*Prevotella*	0.28	0.59	7.92	4.45	8.88	2.446	0.022	0.503


*Firmicutes*	*Bulleidia*	0.00	0.00	1.00	1.98	0.40	0.718	0.373	0.095


	*Butyrivibrio*	0.00	0.02	1.31	1.02	1.26	0.384	0.019	0.178


	*Clostridium*	0.13	0.04	0.10	0.17	0.75	0.255	0.091	0.269


	*Coprococcus*	0.00	0.02	0.23	0.13	0.27	0.072	0.017	0.538


	*Enterococcus*	30.94	0.00	0.00	0.01	0.00	10.386	0.170	0.220


	*Lactobacillus*	32.81	67.95	0.04	0.99	1.09	11.658	0.004	0.119


	*Mogibacterium*	0.00	0.01	2.05	1.85	0.51	0.510	0.207	0.005


	*Moryella*	0.00	0.00	0.46	0.28	0.32	0.140	0.082	0.157


	*Oscillospira*	0.01	0.24	0.19	0.17	0.83	0.289	0.111	0.401


	*Pseudobutyrivibrio*	0.00	0.01	0.56	0.07	1.44	0.363	0.019	0.304


	*Ruminococcus*	0.00	0.19	2.97	2.54	2.43	0.876	0.038	0.100


	*SMB53*	0.16	1.94	12.60	8.71	2.00	4.878	0.677	0.074


	*p-75-a5*	0.00	0.05	1.36	4.50	0.29	0.697	0.144	0.002


*Proteobacteria*	*Escherichia*	2.03	3.67	0.13	2.93	1.18	1.940	0.632	0.909


*Actinobacteria*	*Bifidobacterium*	0.19	0.00	0.00	0.00	0.00	0.065	0.175	0.227


	*Propionibacterium*	1.04	0.02	0.00	0.02	0.01	0.351	0.173	0.225


*Chloroflexi*	*SHD-231*	0.00	0.00	0.59	0.31	0.24	0.125	0.112	0.020


*Fibrobacteres*	*Fibrobacter*	0.00	0.01	0.63	0.71	0.83	0.357	0.076	0.478


*Spirochaetes*	*Treponema*	0.00	0.01	0.61	0.01	0.48	0.267	0.265	0.780


*Verrucomicrobia*	*Akkermansia*	0.00	3.86	0.01	0.03	0.00	1.301	0.293	0.830


	Unclassified	22.01	9.86	63.48	66.90	64.74	10.366	0.001	0.044


	Others (<0.5%)	10.35	9.91	3.77	2.07	11.88	6.153	0.952	0.209

### OTU Diversity and Similarity Analysis

**FIGURE 1:**
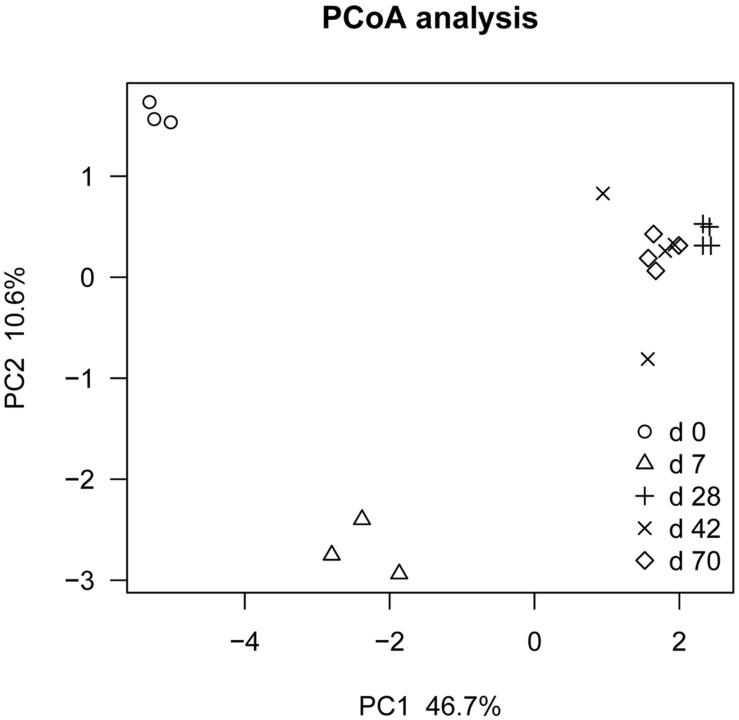
**Principal co-ordinate analysis (PCoA) analysis of ileal bacterial community at different ages using Jackknifed beta diversity of 100 times resampling at a depth of 12,000 sequences in grazing kids (*n* = 18)**.

**FIGURE 2:**
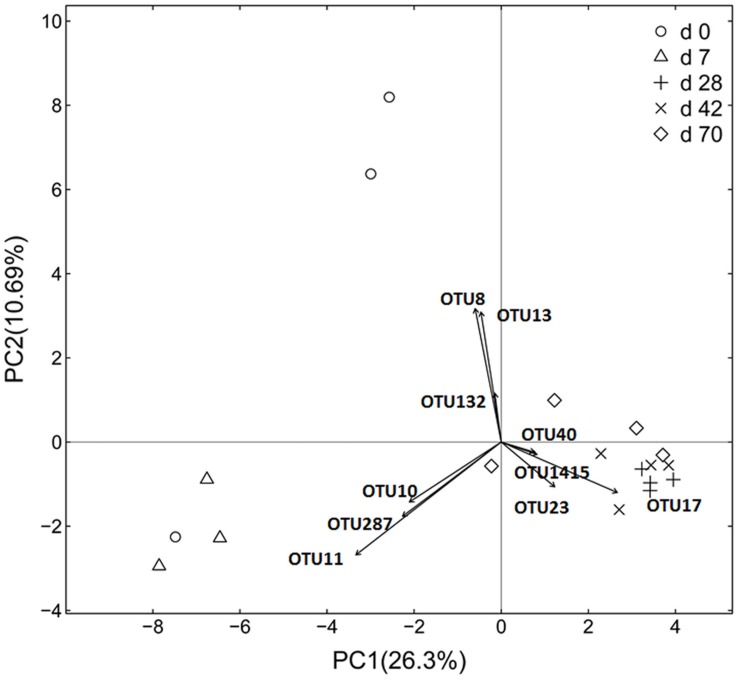
**Transform-based PCA analysis of ileal bacterial community at different ages using the Hellinger distance in grazing kids (*n* = 18)**. The top 10 operational taxonomic units (OTUs) contributing to the separation of samples are displayed on the figure (OTU number). OTU11, *Lactobacillus* genus; OTU10, *Lactobacillus* genus; OTU287, *Lactobacillus* genus; OTU8, *Enterococcus* genus; OUT13, *Halomonadaceae* family; OTU132, *Cloacibacterium* family; OTU17, *Streptophyta* order; OTU23, *SMB53* genus; OTU40, *F16* family; OTU1415, *F16* family.

Principal coordinate analysis based on unweighted UniFrac distance revealed that each age group had its distinct bacteria (**Figure 1**). Transform-based PCA indicated that three OTUs separating day 0 from others were identified as *Enterococcus* genus, *Halomonadaceae* family and *Cloacibacterium* genus (**Figure 2**). Three OTUs discriminating day 7 from others belonged to *Lactobacillus* genus. Furthermore, four OTUs (*Streptophyta* order, *F16* order and *SMB53* genus) accounted for separation of days 28, 42, and 70 from others.

### Correlation between Bacterial Community and Fermentation Parameters

To further investigate the potential role of specific bacteria genera in the consummation of host fermentation capacity, Spearman’s rank correlations were conducted between genus proportion and fermentation parameters. As indicated in **Table 5**, amylase activity negatively correlated with abundances of *Escherichia* (*R* = -0.50, *P* < 0.05), *Pseudobutyrivibrio* (*R* = -0.55, *P* < 0.05) and *Treponema* (*R* = -0.61, *P* < 0.05), while positively correlated with *p-75-a5* abundance (*R* = 0.79, *P* < 0.01). CMCase activity was positively associated with *Enterococcus* abundance (*R* = 0.50, *P* < 0.05), and xylanase activity was positively associated with abundances of *Bacteroides* (*R* = 0.56, *P* < 0.05), *Clostridium* (*R* = 0.50, *P* < 0.05), *Lactobacillus* (*R* = 0.50, *P* < 0.05), and *Propionibacterium* (*R* = 0.63, *P* < 0.05).

**Table 5:** Spearman’s rank correlation coefficiences between ileal bacterial community and fermentation parameters (*n* = 12).

	Amylase	CMCase	Xylanase
*Bacteroides*	-0.08	0.23	0.56ˆ*
*Clostridium*	-0.15	0.21	0.50ˆ*
*Enterococcus*	-0.01	0.50ˆ*	0.46
*Escherichia*	-0.50ˆ*	-0.05	0.01
*Lactobacillus*	0.18	0.40	0.50ˆ*
*Propionibacterium*	0.02	0.24	0.63ˆ*
*Pseudobutyrivibrio*	-0.55ˆ*	-0.17	-0.18
*Treponema*	-0.61ˆ*	-0.06	-0.38
*p-75-a5*	0.79ˆ**	0.00	0.01

*^∗∗^*P* < 0.01, ^∗^*P* < 0.05*.

## Discussion

The microbial community of animal gut has been described as an additional host ‘organ.’ The synergic crosstalk between animals and microbes profoundly influence the maturation and homeostasis of adult life (Hooper and Gordon, 2001). Currently, the colonization process of ileal bacteria in grazing ruminants is poorly understood. In this study, we for the first time characterized the colonization of the ileal bacteria at five developing time points (starting from birth) of grazing goats.

Our results highlighted the notion that ileal bacteria compositions undergo stage-specific changes over development. In mammals, the diversity and quantity of gastrointestinal microbiota increase during development (Combes et al., 2011; Jami et al., 2013; Jiao et al., 2015a; Zhao et al., 2015). In goat ileum, our results demonstrated that total bacteria copy number increased with age, reaching relatively stable values after 1 month, similar to previous observation in goat rumen (Jiao et al., 2015b). PCoA revealed that each age group had its distinct microbiota. Likewise, in human (Jost et al., 2012), piglet (Niu et al., 2015), and cattle (Rey et al., 2014), previous researches indicated that diet, especially intake of breast milk and weaning could exert crucial effect on the microbial community structure. Other potentially important factors that affect the microbial colonization process include the environmental microbial exposure and host physiology (Thompson et al., 2008).

In mammal small intestine, dominant phyla are *Bacteroidetes*, *Firmicutes*, and *Proteobacteria* (Malmuthuge et al., 2014), but the proportion of each phylum is usually fluctuant and affected by multiple factors such as animal species, diet, age and genotype. For new born goats, total bacteria copy number was relatively low in the current study, being consistent with previous observation in calf small intestine (Malmuthuge et al., 2015). For bacteria composition, *Proteobacteria* phylum accounted for a great proportion (23.9%), with *Escherichia* genus constituted 2.0% of total sequences. This was in accordance with previous observations in pig small intestine (Zhao et al., 2015) and bovine rumen (Jami et al., 2013). *Enterococcus* genus also reached high proportion (31.0%) within the first day. Most members of *Escherichia* and *Enterococcus* genera are facultative anaerobic bacteria and can create a reduced environment (Franz et al., 2011; Kalita et al., 2014), allowing the succession establishment of obligate anaerobes to dominant population levels. *Enterococcus* comprises important lactic acid bacteria and produce moderate amounts of lactic acid, which could stimulate growth of lactic acid utilisers and stabilize gastrointestinal pH, and can be used as probiotics to improve animal health (Franz et al., 2011). Moreover, other lactic acid bacteria, *Lactobacillus* also accounted for a great proportion (32.8%), and these lactic acid bacteria could degrade lactose and other oligosaccharides in the milk into acetate and lactate (Walter, 2008), similar to previous trend in the rumen when goat were fed exclusively milk (Jiao et al., 2015b). Another genus needs to be taken into consideration was *Bifidobacterium*, which constituted 0.19% of total sequences in the ileum at day 0. Malmuthuge et al. (2015) has reported the relative higher levels of *Bifidobacterium* (10.4%) within 30 min after birth in neonatal calves.

At day 7, *Proteobacteria* declined drastically from 23.9 to 5.4%. Similarly, in dairy calf rumen, this phylum abundance decreased from 70.4% at day 2 to 16.9% between days 3 and 12 (Rey et al., 2014). *Enterococcus* genus declined significantly to minimal values, consistent with previous observations that *Enterococcus* number showed a continuous decrease from days 3 to 60 of age in the feces of young Creole goats (Draksler et al., 2002). Strikingly, *Lactobacillus* abundance almost doubled at day 7 vs. day 0, accounting for 68.0% of total sequences. Some cultural *Lactobacillus* species found in the gastrointestinal tracts (GIT) have received tremendous attention due to their health-promoting properties, especially in modulating host defenses (Walter, 2008). Moreover, the increase in abundances of two other genera, *Bacteroides* and *Akkermansia* was also observed in this study, and some members of these two were capable of producing mucin-degrading enzymes (Derrien et al., 2004; Troy and Kasper, 2010). These two genera could also be found in a relatively higher proportion in calf rumen fed exclusively milk (Jami et al., 2013).

Bacteria density increased almost one hundred times, and bacterial composition changed significantly after the introduction of forage-based diet, highlighting the role of forage diet on bacterial colonization (in both density and diversity). *Prevotella* genus surged in abundance while *Bacteroides* genus declined drastically in abundance at day 28. A similar compositional change in these two genera has been reported in the gut microbiota of children from rural Africa (De Filippo et al., 2010), which was composed of high abundance of *Prevotella* genus (53%) while low abundance of *Bacteroides* (<3%). African diet is composed mainly of plant fibre, similar to the goat diet (forage) in the present study after day 20. Another noticeable surge in abundance was observed for *Ruminococcus* and *Fibrobacter*, most of which were fibrolytic bacteria, with their abundance increased more than 10 times compared to their values at day 7. This would indicate a promotion in fiber degradation capacity (Jiao et al., 2014a). Other noticeable change lay in the drastic increase in *SMB53* genus (belonging to *Clostridiaceae* family). Most members of this family are capable of consuming mucus- and plant-derived saccharides such as glucose in the gut (Wust et al., 2011), and the pasture forage in the present study after day 20 can provide considerable substrates for its growth.

Investigation on enzyme activities provides insights on carbohydrate digestion capacity of ileal bacteria. Xylanase activity was detected at relatively low values at day 28 and increased thereafter, highlighting the maturation of fiber digestion capacity after forage was offered. Considering cellulolytic bacteria remained high proportion at day 28, there was a lag time between enrichment of cellulolytic bacteria and improvement of fiber degradation capacity. This could be explainable that microbial attachment to fibrous substrates is an important prerequisite of fiber degradation before cellulolytic microbes start to produce enzymes (Saro et al., 2012). Furthermore, age exerted quadratic effect on amylase activity, reaching its peak at day 42. As suggested, on average, 5–20% of starch consumed is digested post-ruminally, with most of that digestion in the small intestine (Huntington, 1997). Since total bacteria number in goat ileum was relatively low, most of the starch entering the ileum should be submitted to the activity of pancreatic a-amylase (Noziere et al., 2010).

Most *Bacteroidetes* phylum members is specialized in the breakdown of complex plant polysaccharides (Xu et al., 2003), and some members of *Clostridiaceae* family are also capable of consuming plant-derived saccharides (Wust et al., 2011). Thus, it is not surprising to uncover positive associations between *Bacteroides* and *Clostridium* abundances and xylanase activity. Furthermore, positive associative relationships have also been observed between some lactic acid bacteria, such as *Enterococcus*, *Lactobacillus* (Walter, 2008), and fiber degradation enzyme activity. Together these results support the notion that a diet high in forage promotes a bacterial structure and metabolite production, which in turn, facilitates fiber digestion capacity of the host.

Inter-individual variation in microbiota is a remarkable characteristic of digestive tracts across animals’ development. In the current study, for instance, *Enterococcus* genus accounted for 92.1% in one sample at day 0, while its abundance in other two samples at day 0 were minor (<1%). Similar inter-individual variation has also been observed in goat and calf rumen (Jami et al., 2013; Jiao et al., 2015a), as well as zebrafish and pig intestine (Niu et al., 2015; Zac Stephens et al., 2016).

## Conclusion

Both the density and diversity of the ileum bacteria in grazing goats increased with increasing age. Bacterial composition changed as goats matured, with *Enterococcus*, *Lactobacillus*, and *Escherichia* being dominant genera at day 0, while *Prevotella*, *Butyrivibrio*, *Ruminococcus*, *SMB53*, and *Fibrobacter* surged in abundance after day 20. These changes were associated with elevated xylanase activity and decreased amylase activity from days 28 to 70. To our knowledge, this is the first study in which bacteria colonization and fermentation capacity in the ileum of grazing goats was related. These findings may help in the development of strategies to guide the formation of health-promoting microbiotas that could then be maintained throughout the life of the host.

## Author Contributions

JJ and ZT designed the research. JJ, JW, CZ, and MW conducted research. JJ and ST analyzed data. JJ and ZT wrote the paper. All authors read and approved the final manuscript.

## Conflict of Interest Statement

The authors declare that the research was conducted in the absence of any commercial or financial relationships that could be construed as a potential conflict of interest.

## Supplementary material

FIGURE S1 **Rarefaction curves (number of OTUs) of ileal bacterial community at different ages in grazing kids (*n* = 18)**.
