# Supplementary figures and images for: Composition of Ileal Bacterial Community in Grazing Goats Varies across Non-rumination, Transition and Rumination Stages of Life
Source: Front Microbiol. 2016 Sep 5;7:1364. doi: 10.3389/fmicb.2016.01364 (PMC5011132; doi:10.3389/fmicb.2016.01364)

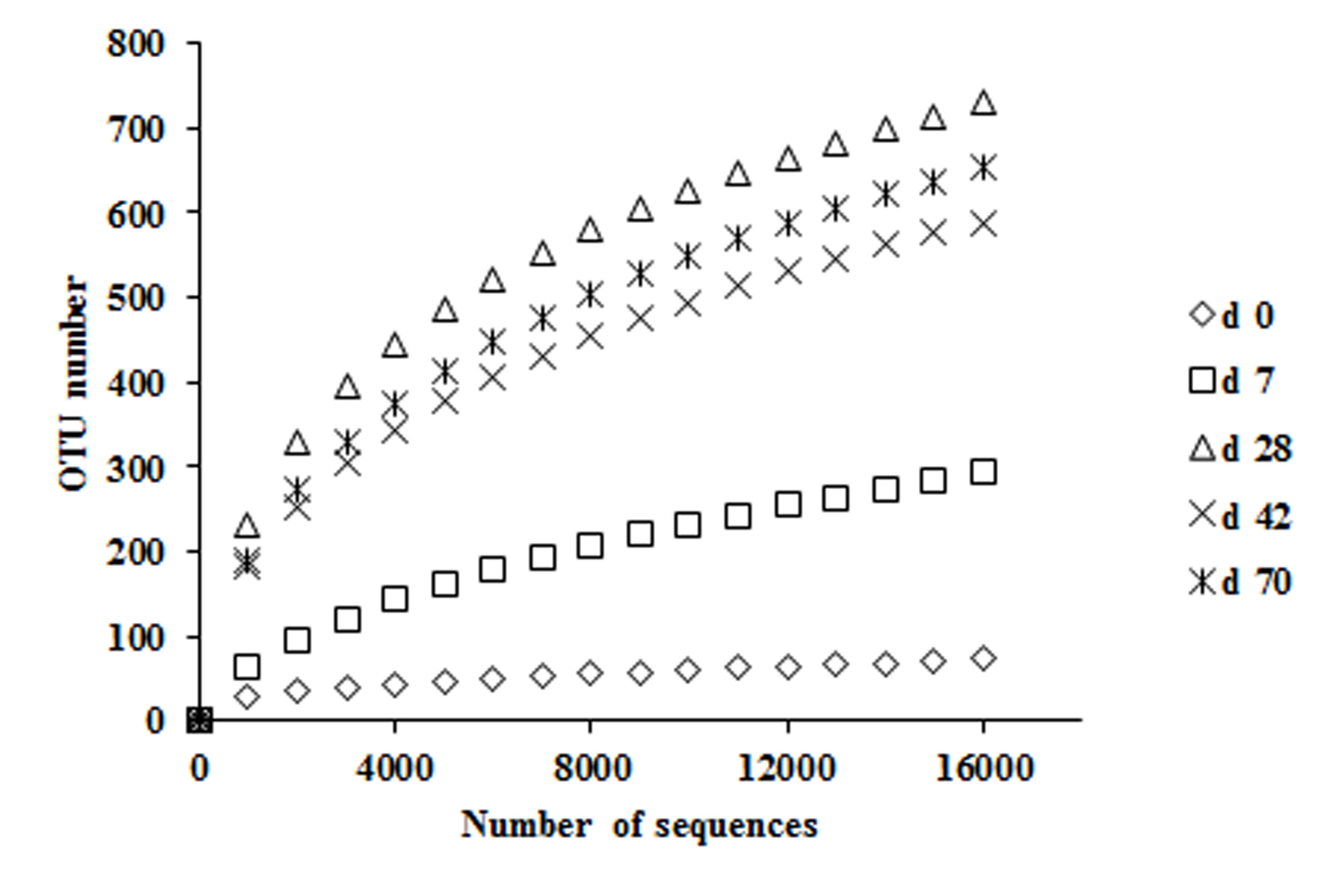

Supplement: FIGURE S1 — Rarefaction curves (number of OTUs) of ileal bacterial community at different ages in grazing kids (n = 18). [file Image_1.JPEG]
